# “Ant” and “Grasshopper” Life-History Strategies in *Saccharomyces cerevisiae*

**DOI:** 10.1371/journal.pone.0001579

**Published:** 2008-02-13

**Authors:** Aymé Spor, Shaoxiao Wang, Christine Dillmann, Dominique de Vienne, Delphine Sicard

**Affiliations:** 1 Université Paris-Sud, UMR de Génétique Végétale, Institut Scientifique de Recherche Agronomique (INRA) / Université Paris-Sud / Centre National de la Recherche Scientifique (CNRS)/ AgroParisTech, Ferme du Moulon, Gif-sur-Yvette, France; 2 Department of Biochemistry and Molecular Biology, Louisiana State University Health Sciences Center, Shreveport, Louisiana, United States of America; Oxford University, United Kingdom

## Abstract

From the evolutionary and ecological points of view, it is essential to distinguish between the genetic and environmental components of the variability of life-history traits and of their trade-offs. Among the factors affecting this variability, the resource uptake rate deserves particular attention, because it depends on both the environment and the genetic background of the individuals. In order to unravel the bases of the life-history strategies in yeast, we grew a collection of twelve strains of *Saccharomyces cerevisiae* from different industrial and geographical origins in three culture media differing for their glucose content. Using a population dynamics model to fit the change of population size over time, we estimated the intrinsic growth rate (*r*), the carrying capacity (*K*), the mean cell size and the glucose consumption rate per cell. The life-history traits, as well as the glucose consumption rate, displayed large genetic and plastic variability and genetic-by-environment interactions. Within each medium, growth rate and carrying capacity were not correlated, but a marked trade-off between these traits was observed over the media, with high *K* and low *r* in the glucose rich medium and low *K* and high *r* in the other media. The cell size was tightly negatively correlated to carrying capacity in all conditions. The resource consumption rate appeared to be a clear-cut determinant of both the carrying capacity and the cell size in all media, since it accounted for 37% to 84% of the variation of those traits. In a given medium, the strains that consume glucose at high rate have large cell size and low carrying capacity, while the strains that consume glucose at low rate have small cell size but high carrying capacity. These two contrasted behaviors may be metaphorically defined as *“ant”* and *“grasshopper”* strategies of resource utilization. Interestingly, a strain may be *“ant”* in one medium and *“grasshopper”* in another. These life-history strategies are discussed with regards to yeast physiology, and in an evolutionary perspective.

## Introduction

Adaptation of species to environment is characterized by an increase of fitness, classically defined as the expected number of offspring of an individual [1]. In living organisms, three life-history traits play an essential role in fitness: the reproduction rate, the carrying capacity and the body size [2]. At the population level, the reproduction rate, estimated by the intrinsic growth rate (*r*), quantifies how much a population can grow between successive time periods. The carrying capacity (*K*), maximum size of the population that can be supported upon the available resources, reflects the expansion ability of a species [3], [4]. Finally, at the individual level, the body size is related to lifespan, home range size and other life-history traits [5], [6].

Variation of life-history traits has been reported in a wide range of plant and animal species [2], [7]. More importantly, negative correlations between life-history traits, in particular between *r* and *K*, as well as between body size and *K*, have been found both in natural populations [6], [8]–[11] and in artificial selection populations [12], [13]. This suggests that selection does not optimize these fitness traits separately. The available resources are shared between maintenance, size growth and reproduction. This “Partition of Resources Model”, in which resource allocation to one trait means deprivation to another, has been proposed as the main explanation for trade-offs between life-history traits [7], [14]. For instance limitations in common internal metabolites may result in competition between reproduction and maintenance pathways [15]. Thermodynamic constraints also lead to trade-offs, as it is the case between yield and rate of ATP-producing pathway [16], [17].

In a given environment, differences in life-history strategies between populations or species have necessarily a genetic basis [7], whereas changes in environmental parameters may affect life-history traits of a given population/species [18]. Finally genotype-by-environment interactions can also account for variability in life-history strategies [18]. Actually in natural populations the origin of the variation in trade-offs cannot easily be determined, because no control of the factors that might affect life-history traits exists. This is the “missing variable” problem [7]: in non-controlled conditions one cannot be sure that all relevant variables are included in the analysis. For instance, a study on the *Campanula* genus in Greece has revealed a shift between the *r*- and *K*-strategies among species growing at different elevations [11], but whether this shift depends on the species or the environment is unknown. By contrast selection experiments can give information on possible genetic bases of the trade-offs, though whether the trade-offs change with environment has not been studied much. In *Drosophila melanogaster* a genetic trade-off between *r* and *K* has been detected [12], [19]–[21], but experiments in *Escherichia coli* gave contrasted results. While Luckinbill *et al.* failed to show any [22], [23], Novak *et al.*, using populations of *E. coli* from the long-term evolution experiment, detected a trade-off between growth rate and yield within 3 over 4 tested evolved populations [24].

Surprisingly few studies have been carried out to estimate correlations between life-history traits in two or more environments, which still represents the direct experimental method to untangle the genetic and environmental components of the trade-off variation, and possible genetic-by-environment interactions. In addition, usually little attention is paid to the biochemical features of the life-history traits and trade-offs. Understanding the causes of the variability and correlations of life-history traits requires the analysis of the rate of resource uptake, which depends both on the amount of resources in the environment and on the activity of enzymes involved in the uptake. Increasing resource uptake rate might be efficient for fast growth and reproduction in a rich environment, but can lead to fast resource exhaustion in poor environments and hence in increased mortality before reproduction and/or expansion. Thus the resource consumption rate appears as a key parameter to account for variation in life-history traits, and more specifically genetic-by-environment aspects.

Although there is a huge literature on plant and animal life-history strategies, there are few reports on these questions in microorganisms [24]–[27]. Microorganisms are convenient models for evolutionary ecology because they have usually a short generation time, a large population size, are mostly unicellular with no developmental differentiation and are easy to manipulate in the laboratory.

The yeast *Saccharomyces cerevisiae*, a common biological model in genetics and physiology, has only recently been chosen as a model in evolutionary genetics [28], [29], and to our knowledge has never been used to study life-history theories. In this work, we have analyzed the plastic and genetic components of life-history traits in *S. cerevisiae* and have analyzed the influence of resource uptake rate on the trade-offs between those traits. In a collection of twelve *S. cerevisiae* strains coming from various industrial and geographical origins and grown in three culture media differing in glucose concentrations, we analyzed the variability of growth rate, carrying capacity and cell size. For each strain in each medium, we also measured the glucose consumption rate per cell, in order to assess the possible role of this parameter in the traits' variation. We showed that both plastic and genetic factors strongly affect the yeast's life-history strategies. Changes in the glucose content of the medium results in a shift of the balance between carrying capacity and growth rate, and between growth rate and cell size, while in a given medium the strains may differ to a large extent for carrying capacity, cell size and glucose consumption rate, making it possible to define what we called the *“ant”* and *“grasshopper”* life-history strategies, inspired by the behaviour of these animals in the well known fable popularized by Aesop.

## Results

### Genetic and plastic components of traits' variation

Three life-history traits, the growth rate (*r*), the carrying capacity (*K*) and the cell size (*S*), along with glucose consumption rate *per cell* (*J_T_*_50_), were measured in twelve *S. cerevisiae* strains coming from three industrial origins (bakery, brewery and vinery; see table 1) and grown in three culture media (0.25%, 1% and 15% glucose). An analysis of variance was performed for every trait (Table 2).

**Table 1:** Collection of strains of *Saccharomyces cerevisiae*

Accession number*	Geographical origin	Industrial origin
154	Russia	Vinery
157	Spain	Vinery
328	England	Vinery
479	Italy	Vinery
402	Japan	Brewery
221	Czech Republic	Brewery
227	The Netherlands	Brewery
804	Germany	Brewery
215	New Zealand	Bakery
319	France	Bakery
324	Vietnam	Bakery
646	United States	Bakery

* Strains were obtained from the CIRM-Levures (Centre International de Ressources Microbiennes, Thiverval-Grignon, France). They are identified by their accession number in this collection.

**Table 2:** Mean trait value and ANOVAs' results for carrying capacity (log*K*), glucose consumption rate (−log*J_T_*_50_), growth rate (*r*), and cell size (−log*S*).

	df	log*K*	−log*J_T_*_50_	*r*	df	−log*S* ^(a)^
***Mean trait value***		17.8	19.89	0.0097		17.84
***Total sum of squares***		37.18	43.8	2.23 10^−3^	34	11.12
***Percent sum of squares*** ^(b)^						
Origin	2	7.78**	9.16**	3.34*		6.38**
Medium	2	75.49**	33.80**	73.90**		79.91**
Strain(origin)	9	2.64	1.55	3.83		/
Block(medium)	7	0.83**	11.44**	2.42**		/
Medium*origin	4	2.58	24.39**	2.01		5.91**
Medium*strain(origin)	18	8.26**	14.34**	6.51**		/
Residual	73	2.42	5.32	7.99		7.80
*Total*	115					

For cell size the ANOVA model is different from the one used for the other traits (see Material and Methods).

df : degree of freedom. (a) As there was only one experimental repetition for cell size, the residual variation also contains differences between strains. (b) Type I sums of squares are expressed as a percentage of the total sum of squares. Significance was assessed under a mixed model with Strain(origin) and Medium*strain(origin) as random effects.

* Significant at 5%.

** Significant at 1%.

#### Medium effect

**Figure 1:**
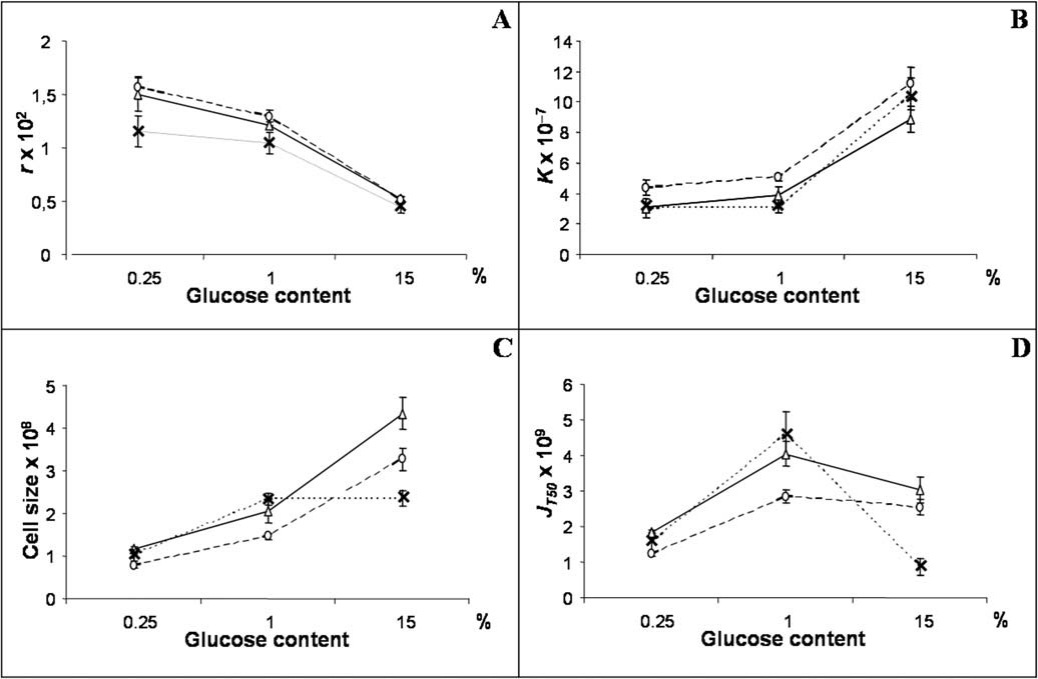
Variation of intrinsic growth rate *r* (A), carrying capacity *K* (B), cell size (C) and glucose consumption rate *J_T50_* (D) in three culture media differing for glucose content. Each point represents the mean value of the trait for each industrial origin in each culture medium with the standard error associated. Vinery strains are represented by circles, brewery ones by crosses and bakery ones by triangles.

For all traits, the medium effect was highly significant (Table 2 and Figure 1), and accounted for the most important part of total variation (from ∼34% for *J_T_*_50_ up to ∼80% for *S*). The mean growth rate significantly decreased when the resources of the medium increased (Figure 1A). By contrast, the mean carrying capacity increased when the resources increased (Figure 1B): it was about three fold higher in the glucose rich medium (15%) than in the other media. Mean cell size also increased with resources (Figure 1C). More surprisingly, mean glucose consumption rate reached its maximum value in the 1% glucose medium (Figure 1D).

#### Industrial origin effect

The industrial origin effect was significant for all traits, and explained from 3.34% (for *r*) to 9.16% (for *J_T_*_50_) of the total variation (Table 2). The ranking order of the industrial origins changed according to the trait. Bakery and vinery strains had similar growth rates (*p* = 0.4259), higher than the brewery one (*p*<0.0001) (Figure 1A). Bakery and brewery strains showed similar carrying capacity (*p* = 0.1161), smaller than vinery ones (*p*<0.0001) (Figure 1B). Bakery strains had higher cell size than brewery (*p* = 0.0608) and vinery ones (*p*<0.0001) (Figure 1C). They had also a higher glucose consumption rate (*p*<0.0001) than brewery and vinery strains (Figure 1D).

#### Medium-by-origin interactions

No significant medium-by-origin interaction effect was detected for the growth rate and the carrying capacity. By contrast, significant interaction effects were found for glucose consumption rate (24.39% of the total sum of square) and for cell size (5.91%) (Table 2). For both traits, differences between industrial origins depended on the glucose concentration in the medium. Mean values increased when going from 0.25% to 1% glucose, but reaction norms crossed between 1% and 15%. Brewery strains did not increase in size and decreased their glucose consumption rate in 15% glucose, while for bakery and vinery strains cell size increased and glucose consumption rate remained constant (Figure 1C and 1D).

#### Strain effects and medium-by-strain interactions

The strain effects within origins were never significant, but the medium-by-strain interactions were significant for all traits tested, with a percentage of total variation explained as high as 14.34% (for *J_T_*_50_) (Table 2). This means that for a given industrial origin, the ranking order of the strains is clearly medium-dependent. In addition the percent of variation explained by the sum of the medium-by-genotype interactions (‘medium-by-strain(origin)’ plus ‘medium-by-origin’) was consistently higher than the one explained by the main genetic effects (‘origin’ plus ‘strain(origin)’) (Table 2). For the glucose consumption rate, it even exceeded the main effect of the medium.

### Correlations between life-history traits

Correlations between traits were studied using the average trait value of each strain in each medium as variable. They were analyzed both globally taking all the data together (total phenotypic correlations) and within each medium (genetic correlations within media).

#### Total phenotypic correlations

**Figure 2:**
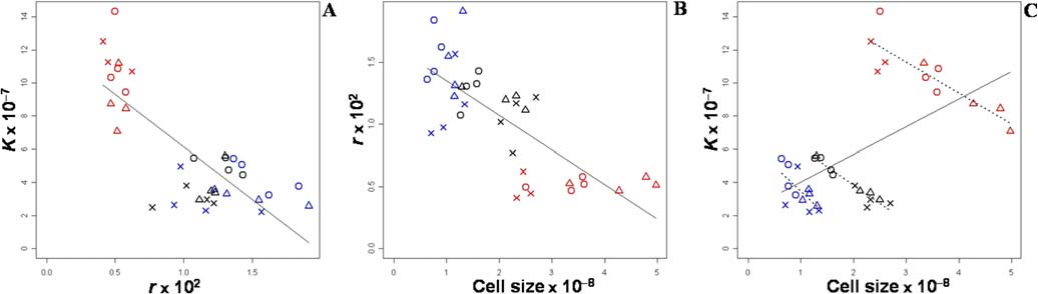
Trade-offs between carrying capacity *K* (A), growth rate *r* (B), cell size (C) across culture media. Each point corresponds to the mean value of a strain in a culture condition. Blue, black and red colors correspond respectively to 0.25%, 1% and 15% glucose. Vinery strains are represented by circles, brewery ones by crosses and bakery ones by triangles.

Pairwise rank phenotypic correlations between the three life-history traits were all significant (Figure 2). A clear trade-off between growth rate and carrying capacity was observed (*r_s_* = −0.644, *p*<0.0001) (Figure 2A). There were two separated groups: one corresponded to the strains grown in the glucose rich medium (15%), with low *r* and high *K* values, and the other to the strains grown in the more restrictive media (0.25% and 1% glucose), with small *K* and high and variable *r* values. In addition, a trade-off between growth rate and cell size was found (*r_s_* = −0.731, *p*<0.0001). As glucose increases in the medium, the growth rate decreases and cell size increases (Figure 2B). Finally there was a positive correlation between cell size and carrying capacity (*r_s_* = 0.507, *p* = 0.002) (Figure 2C). All these correlations were mainly related to the strong effect of media on the variation of the life-history traits (Figure 2).

#### Genetic correlations within media

Within media, no correlation was found between carrying capacity and growth rate, or between growth rate and cell size (Figure 2A and 2B). On the other hand, whereas cell size and carrying capacity are globally positively correlated over all media, they displayed a trade-off within each medium, as shown by the significant negative correlations (*ρ* = −0.65, *ρ* = −0.96 and *ρ* = −0.87, *p*<0.01, for 0.25%, 1% and 15% glucose respectively) (Figure 2C).

### Relationship between glucose consumption rate per cell and life-history traits

**Figure 3:**
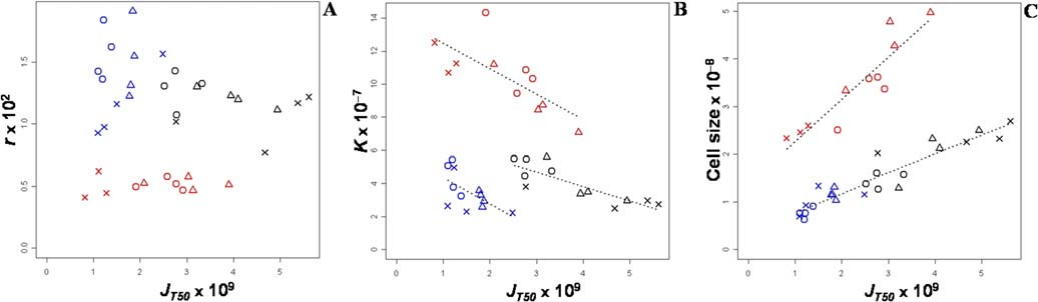
Correlations between glucose consumption rate and growth rate per cell (A), carrying capacity (B) and cell size (C). Each point corresponds to the mean value of a strain in a culture condition. Blue, black and red colors correspond respectively to 0.25%, 1% and 15% glucose. Vinery strains are represented by circles, brewery ones by crosses and bakery ones by triangles.

Surprisingly, the glucose consumption rate per cell did not show any correlation with growth rate, neither within media, nor globally (Figure 3A). By contrast, within each medium, significant negative correlations were found between glucose consumption rate and carrying capacity, and positive ones between glucose consumption rate and cell size (Figure 3B and 3C). In the 0.25%, 1% and 15% glucose media, the glucose consumption rate accounted for 37%, 70% and 54% of the carrying capacity variation, respectively, and for 50%, 74% and 84% of cell size variation, respectively (*p*<0.05).

**Figure 4:**
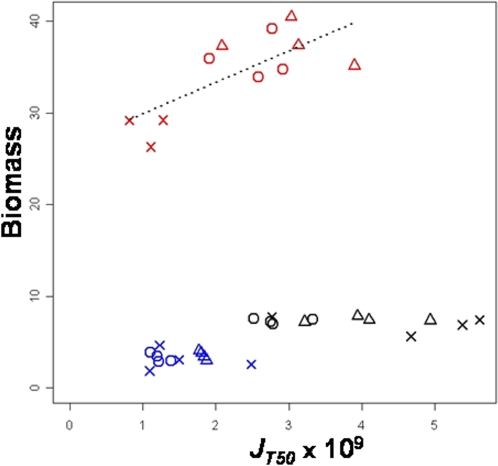
Relationship between biomass and glucose consumption rate. Biomass is defined as the product of carrying capacity by cell size. Each point corresponds to the mean value of a strain in a culture condition. Blue, black and red colors correspond respectively to 0.25%, 1% and 15% glucose. Vinery strains are represented by circles, brewery ones by crosses and bakery ones by triangles.

The negative correlation between carrying capacity and cell size for a given amount of resource and their opposite responses to variations of glucose consumption rate are consistent with the trade-off between those traits. Glucose may be allocated either to an increase of population size or to cell size. To further analyze the resource allocation we studied the biomass at the end of the culture, defined as the product of carrying capacity by cell size. The glucose consumption rate was not related to biomass in 0.25% and 1% glucose, indicating that glucose is equivalently allocated either to cell size or to carrying capacity in these media (Figure 4). A positive correlation was observed in 15% glucose (*ρ* = 0.74, *p*<0.01) indicating that, when resources are abundant, the increase of cell size is not fully compensated by the decrease of population size.

Altogether these results revealed that yeast strains range between two typical strategies for resource utilization. On the one hand some strains have a low glucose consumption rate per cell, a small cell size and a high carrying capacity. On the other hand other strains have a high glucose consumption rate, a large cell size but a low carrying capacity. In reference to the famous Aesop's (620–560 BC) fable, we propose to call these two genetically contrasted behaviors *“ant”* and *“grasshopper”* life-history strategies, respectively.

**Figure 5:**
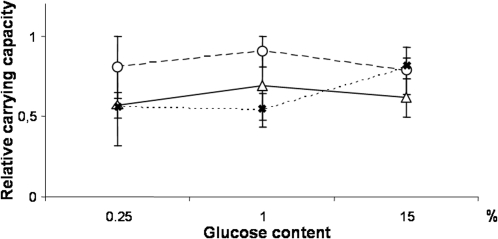
Variation of the relative carrying capacity in the three culture media. In each medium, the relative carrying capacity of each strain was calculated as the ratio of its carrying capacity to the highest strain's carrying capacity in this medium. Each point represents the mean value of the relative carrying capacity for each industrial origin in each culture medium with the standard error associated. Vinery strains are represented by circles, brewery ones by crosses and bakery ones by triangles.

The high medium-by-genotype interactions observed for the glucose consumption rate indicates that a given strain can behave as a *“grasshopper”* in one medium and as an *“ant”* in another. This is for example clearly the case for three brewery strains and one bakery strain which are *“ants”* in 15% glucose and *“grasshopper”* in 1% glucose (Figure 3B). More globally, the vinery strains have the highest mean relative carrying capacity and behave as *“ants”* in all glucose conditions, but the brewery strains tend to be *“grasshoppers”* in low glucose conditions (0.25% and 1%) and *“ants”* in 15% glucose, while the bakery strains display an intermediary behavior (Figure 5). It is important to note that despite these medium-by-genotype interactions, there is always a negative correlation between carrying capacity and glucose consumption rate.

## Discussion

### Plasticity of the life-history traits

The life-history traits, carrying capacity, intrinsic growth rate and cell size were strongly affected by the glucose content in the culture medium, with obvious trade-offs between carrying capacity and growth rate, and between growth rate and cell size. When resources were abundant (15% glucose), all yeast strains were growing slowly (small *r*), but reached a large population size (*K*) and had bigger cells. On the contrary when glucose was limited in the medium, the strains had higher growth rate, and displayed smaller carrying capacity and cell size. A possible biochemical mechanism for those trade-offs can be proposed. In rich medium, the large amount of extracellular glucose causes a hyperosmotic stress to the cells, which activates the Ras-cAMP pathway [30]. This activation is transducted by the cAMP, the accumulation of which leads to two responses: (i) it stimulates cAMP-dependent protein kinase, which is involved in posttranslational regulation of glycolytic enzymes, and in turn activates glycerol accumulation that counteracts the effect of hyperosmotic stress [31]; (ii) cAMP represses the cyclins CLN1 and CLN2 that are implicated in cell cycle initiation [32], [33]. In addition glucose is known to repress the CLN1 promoter [34]. As a consequence of the delay in cell cycle and of the accumulation of glycerol, cell size increases and intrinsic growth rate decreases when external glucose concentration increases.

Selection could have lead to plasticity for life-history traits, as an adaptive response to unstable or stressing environments that yeasts may encounter in natural or industrial conditions [35]. If plasticity were adaptive, a second step of adaptation to new environments would be the conversion of non-heritable environmentally induced variation into heritable variation [36], by the process often referred to as Baldwin Effect, or genetic assimilation [37], [38]. Experimental evolution would allow to test this scenario, by growing strains in different glucose conditions over many generations and checking if the evolved populations have a reduced plasticity.

### Genetic variability of the life-history traits

The carrying capacity, the growth rate and the cell size − as well as the glucose consumption rate −, proved to be genetically variable among the twelve yeast strains. In particular the industrial origins were clearly distinct to each other, whatever the trait considered. This could be explained by human selection. In the 15% glucose medium, where a high amount of ethanol is produced, vinery and brewery strains reach a higher carrying capacity than bakery ones, indicating a possible adaptation to high levels of ethanol. The higher cell size of bakery strains could also be explained by selection. In bread dough, yeasts may counteract the effect of high osmotic pressure by active uptake of osmolytes, which results in larger cell size [39], [40]. Differences in glucose consumption rate are more difficult to explain in terms of human selection. Historical records indicate that development of brewing industry was accompanied by exchanges between brewers and bakers since the 19^th^ century. Phylogenies based on neutral genomic regions tend to cluster the bakery and brewery strains, away from the vinery ones [41]–[43], and microsatellite analysis revealed that bakery and brewery strains share more alleles than they do with vinery strains [44]. In 0.25% and 1% glucose media, the bakery and brewery strains have well close glucose consumption rates, but not in 15% glucose where the glucose consumption rate of the brewery strains drop steeply. Again, selection experiments could help in analyzing and understanding the effect of human selection on this trait.

### Genetic correlations between life-history traits

The only significant correlation within media was observed between carrying capacity and cell size, with a marked trade-off. In a given medium, the large-cell strains had low carrying capacity and vice versa, which resulted in low genetic variability of the biomass, defined as the product of the cell size by the carrying capacity.

Growth rate was correlated neither to carrying capacity, nor to cell size. Of course this does not mean that these traits are physiologically independent. It has been shown that during yeast growth, cell cycle (i.e. *r*) and cell growth are coordinated [45], and the molecular mechanisms of this coordination are well described [46]. Our result suggests that cell size and intrinsic growth rate can, at least in part, evolve independently of one another.

The relationships between the glucose consumption rate and the life-history traits within media gave partly unexpected results since we did not detect any correlation between intrinsic growth rate and glucose consumption rate. In *Escherichia coli* a hyperbolic ascending relationship was found between the activities of β-galactose permease and β-galactosidase and growth rate for different strains grown on lactose [47]. Our result suggests that human selection has uncoupled the growth rate and the rate at which resources are consumed in *S. cerevisiae*, probably because strains with a high metabolism rate and a quite low division rate have a better yield of fermentation in industry.

By contrast, we found a high positive correlation between cell size and glucose consumption rate. It has been shown that glucose consumption rate is correlated to the number of glucose transporters on the cell surface [48]. Thus the higher glucose consumption in large cells could simply result from the larger number of transporters. Note that if the number of transporters were strictly proportional to the cell surface, the relationship should not be linear, but should follow the relationship 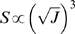. However we do not have enough data to decide between this non-linear and the linear relationship.

We found also highly significant negative linear correlations between carrying capacity and glucose consumption rate, which was expected given the trade-off between carrying capacity and cell size. Altogether, these results show that two opposite genetically-based life-history strategies can be defined within the yeast species. In a given culture medium, some strains (the *“grasshoppers”*) consume glucose faster, reach a bigger cell size at the expense of the carrying capacity, whereas other strains (the *“ants”*) consume slowly glucose, reach a smaller cell size, but have a higher carrying capacity. It is also enlightening to consider the biomass of the strains at the end of the culture. The *“grasshoppers”*, with glucose consumption rates up to 4 times higher than the *“ants”*, display similar or only slightly higher biomass values. This result shows the accuracy of the balance between carrying capacity and cell size, which is in a large extent accounted for by the glucose consumption rate.

### Tragedy of the commons

The existence of different life-history strategies could be related to metabolic constraints. If individuals have access to a common resource, they can either exploit it rapidly (increase the energy produced per unit of time) or efficiently (increase the amount of energy produced per unit of resource consumed). Evolutionary models argue that this trade-off leads to a social conflict in which individual reproductive rate is maximized by consuming resource quickly and population fitness is increased by consuming resource at a higher efficiency.

**Figure 6:**
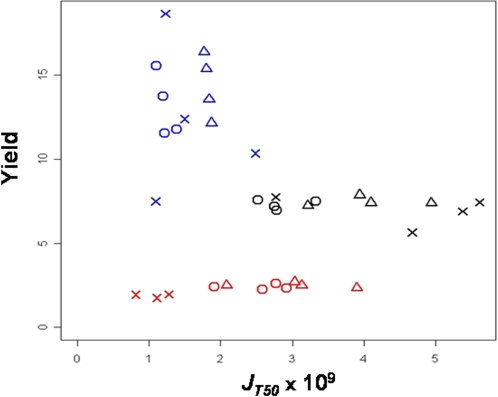
Relationship between yield and glucose consumption rate. Yield is defined as the ratio of the biomass (product of carrying capacity by cell size) to the initial glucose amount in the medium. Each point corresponds to the mean value of a strain in a culture condition. Blue, black and red colors correspond respectively to 0.25%, 1% and 15% glucose. Vinery strains are represented by circles, brewery ones by crosses and bakery ones by triangles.

Experimental data showing a trade-off between yield and resource consumption rate were found among yeast species evaluated in different conditions [49] or for a single strain in different conditions [50] (reviewed in [27]). We also found a plastic trade-off (*r_s_* = −0.74, *p*<0.0001) between glucose consumption rate and yield (calculated as the ratio of biomass to initial glucose concentration) when we considered the data from the two limited glucose media (0.25% and 1%), in the range of resources similar to the one used in previous studies [49], [50]. But we did not detect any trade-off when adding the rich glucose medium, which suggests that the trade-off between yield and rate depends highly on the environment tested (Figure 6).

No genetic correlations between glucose consumption rate and yield were detected in any medium. Instead of the rate/yield relationship, the genetic trade-off between *K* and cell size appears to be more relevant for studying social conflicts: the *“ants”* which consume resource slowly would save resources for the good of the whole population (cooperators) while the *“grasshoppers”* which consume the resources fast, would keep the resource selfishly inside the cell. Additional experiments are needed to explore the possible social conflict between *“ants”* and *“grasshoppers”*.

### The genetic and plastic basis of carrying capacity

Carrying capacity is classically defined as the maximum population size that a given environment can support given finite resources [51]. In ecology, when it is expressed in mathematical models, *K* is often defined as a fixed parameter, because differences among individuals within a species are assumed to be negligible. We showed that this parameter depends not only on the food supply of the environment but also on the genotype of the strains, and that resources consumption rate is a key determinant of the carrying capacity *via* the trade-off with cell size. The genetic variability of the carrying capacity, as well as its relationship with resources consumption rate, should therefore be taken into account in population dynamics modeling.

### Further prospects

From this study, genes involved in resource uptake rate appear as good candidates for studying the genetic basis and the evolution of life-history traits. Identifying them and understanding the underlying physiological mechanisms would bring new insights in evolutionary ecology, and would open possible ways for genetic improvement of industrial strains.

## Materials and Methods

### Biological material

Twelve industrial strains of *S. cerevisiae* coming from the CIRM-Levures (Centre International de Ressources Microbiennes, Thiverval-Grignon, France) were chosen: four brewery strains, four vinery strains and four bakery strains, which came from different countries (Table 1). For each strain, a reference stock is conserved at −80°C in our lab. For each growth condition and each replication a single new colony was isolated from the reference stock.

### Culture conditions

Growth kinetics were realized in fermentation conditions (anaerobic), in liquid medium containing 1% yeast extract (DIFCO) and 0.25%, 1% or 15% glucose. We chose contrasted culture media to reflect a wide range of possible environmental conditions, starting with 0.25% glucose because from this concentration the maximal expression of glycolytic genes is achieved [52]. The cultures were incubated at 30°C under 200 rpm agitation. In these conditions, yeasts are exclusively dividing by mitosis. After an overnight culture, 1 to 5.10^6^ cells were put into 50 mL fresh medium (30°C, 200 rpm). Every hour, 300 µL of each culture was taken, 200 µL to estimate population size and 100 µL to quantify glucose consumption. Each growth kinetics was repeated independently from two to four times to account for experimental variation. Each replication was started with a new colony from the reference stock. As it was not possible to manipulate all strains*medium combinations at the same time, we used a split-splot experimental design. One replication consisted in the twelve strains in one culture medium.

### Estimating cell number and cell size

In *S. cerevisiae*, one way to follow the evolution of population size is to measure the Optical Densities (OD) at 600 nm, which depends on the cell number and cell size:
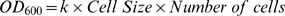
where *k* in an extinction coefficient. The relation between *OD*_600_ and the number of cells was first determined by counting the cells with a Burker's cell. Actually this counting includes both living and dead cells, which do not have the same optical properties. So to follow the change of the number of living cells in the culture over time, samples of each culture were taken during the kinetics and diluted, using the previous counting, to obtain about 100 cells in 100 µL. They were then plated on Petri dishes and incubated at 30°C, and the Colony Forming Units (CFU), *i.e.* the number of living cells forming colonies, were counted. For each of the twelve strains and for each culture medium, the mean cell size (up to the proportionality constant *k*) was estimated as the slope of the regression line of *OD*_600_ on the number of living cells. This was done for the first replication using the model:
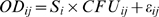
where *S_i_* is the cell size for the strain*-*medium combination *i* (*i* = 1, …, 36), *j* is the time point index, and *ε_ij_* is the residual. We considered the estimate of *S_i_* as characteristic of the strain*-*medium combination *i*, and further used it in the other replications to estimate the cell numbers as:
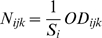
where *k* is the index of the replication, *i* and *j* having the same definition as above.

### Estimating population dynamics parameters

The changes over time of the estimated living cell number were analyzed using two population dynamic models, the Logistic model:
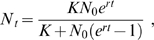
(1)and the Gompertz model:
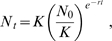
(2)where *N_t_* is the population size at time *t*, *K* is the carrying capacity (equivalent to the maximum population size), *N_0_* is the initial population size and *r* is the intrinsic growth rate (equivalent to the maximum rate of increase of the population, in min^−1^). These two models differ by the way they incorporate competition between individuals. Fitting the growth kinetics with these models allowed estimating *K* and *r*. The estimates from the Logistic model were used because this model best fitted the experimental data, as attested by the lowest residual sums of squares (data not shown). For each replication and each strain-medium combination, about 15 time points were used to estimate *r* and *K*.

In each medium, the relative carrying capacity of each strain was calculated as the ratio of its carrying capacity to the highest strain's carrying capacity in this medium. The maximum relative carrying capacity is thus 1.

### Glucose consumption rate

Glucose consumption rate was estimated by following the evolution of glucose concentration in the medium through time. These measures were made using an enzymatic kit (rBiopharm) composed of the two enzymes hexokinase and glucose-6-phosphate dehydrogenase, which catalyze the following reactions:
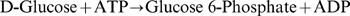


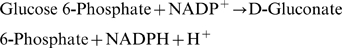



The NADPH formed by this reaction, measured by spectrophotometry at 365 nm, is stoechiometrically equal to the glucose consumed over time.

These extracellular glucose concentrations were adjusted to the Hill equation:
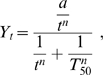
where *Y_t_* is the glucose concentration in the medium (g.L^−1^) at time *t*, *a* is the initial glucose concentration, *n* is the Hill coefficient, and *T*_50_ the time after which the glucose concentration in the medium is half the initial one. Adjustment of the data to the Hill equation gives estimates of *a*, *T*_50_ and *n*.

The glucose consumption rate per cell is given by:
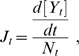
where 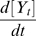 is the rate of glucose consumption and *N_t_* the population size at time *t*. The glucose consumption rate, reported to one liter of medium, is expressed in g.min^−1^.cell^−1^.

As differences of glucose consumption rates between strains were the same over time (tested by regression analysis, data not shown), we chose to consider only the glucose consumption rate at the time after which 50% percent of the initial glucose amount has been consumed. This value is noted *J_T_*_50_.

### Statistical analysis

Variation of each variable among media and strains was analyzed with a mixed model of analysis of variance:
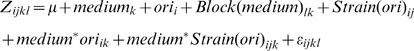
(3)where *Z* is the variable (*r*, *K* or *J_T_*_50_), *medium* is the medium effect (*k* = 1, 2, 3), *ori* is the industrial origin effect (*i* = 1, 2, 3), *Block(medium)* is the random block effect (experimental repetition) within culture medium (*l* = 1, 2, 3, 4), *Strain(ori)* is the random strain effect within industrial origin (*j* = 1, 2, 3, 4), medium*ori (fixed) and medium*Strain(ori) (random) are interaction effects and *ε* is the residual error.

For cell size *S*, as we had only one block we used the following model of analysis of variance:
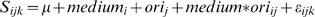
where *medium* is the medium effect (*i* = 1, 2, 3), *ori* is the industrial origin effect (*j* = 1, 2, 3) and medium*ori is the interaction effect.

For each trait, normality of residual distribution was assessed by a Kolmogorov-Smirnov test, and homogeneity of residual distributions was studied. For *S*, *K*, and *J_T_*_50_, a logarithmic transformation proved to be necessary to have the residues distributed normally and homogeneously.

### Comparisons of means between industrial origin

The average performances of strains of origins *i* and *i*' in medium *k* were compared using the least square estimation of *Y_ik_* obtained by averaging the phenotypic value over strains from the same origin and replications. Least square mean and variance estimates were obtained from PROC GLM SAS Procedure. Significance of differences between means was assessed using Tukey HSD method.

### Correlations between traits

The analysis of correlations between traits was carried out on the trait values averaged over replicates of each strain grown in each media. We focused on the variability between strains, neglecting their industrial origin and considering the strain effect as a random effect.

#### Total phenotypic correlations (over all media)

The Multivariate ANalysis Of VAriance general model (MANOVA) is of the kind:
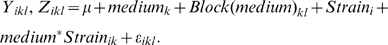



However, as several effects were confounded (notably *Block (medium)*, *medium*, *Strain* and Strain*medium effects), we decided not to estimate all variances and covariances components. Instead, pairwise total phenotypic correlations were studied using Spearman's rank correlations (*r_s_*).

#### Genetic correlations (within each medium)

A separate analysis was conducted in each medium to estimate genetic correlations. Within each medium the two sources of variation were *Strain* and *Block* effects and the MANOVA model should be:
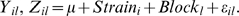



Instead of computing classically the genetic correlations, we chose to compute correlations between traits from strain mean values, because this method has been shown to provide more powerful estimates than the former [53]. However, when replications were available (*r*, *J_T50_*, *K*), we checked that the genetic correlations obtained from a MANOVA (SAS PROC GLM) were of the same sign and of the same order of magnitude as phenotypic correlations.
